# Burnout among Nurses in a Nigerian General Hospital: Prevalence and Associated Factors

**DOI:** 10.5402/2012/402157

**Published:** 2012-04-29

**Authors:** Victor Olufolahan Lasebikan, Modupe Olusola Oyetunde

**Affiliations:** ^1^Department of Psychiatry, College of Medicine, University of Ibadan, P.O. Box 31395, Ibadan, Nigeria; ^2^Department of Nursing, College of Medicine, University of Ibadan, PMB 5116, Ibadan, Nigeria

## Abstract

*Objective*. To evaluate the prevalence and associated factors of burnout among nurses in a Nigerian general hospital. *Methods*. A total sampling method was utilized. *Measurements*. Burnout was evaluated using the Maslach Burnout Inventory; GHQ-12 was used to determine the presence of psychiatric morbidity. *Results*. A high level of burnout was identified in 39.1% of the respondents in the area of emotional exhaustion (EE), 29.2% in the area of depersonalization and 40.0% in the area of reduced personal accomplishment. Multivariate analysis showed that doctor/nurse conflict (OR = 3.1, 95% CI: 1.9−6.3), inadequate nursing personnel (OR = 2.6, 95% CI: 1.5–5.1), and too frequent night duties (OR = 3.1, 95% CI: 1.7–5.6) were predictors of burnout in the area of EE, doctor/nurse conflict (OR = 3.4, 95% CI: 2.2–7.6) and too frequent night duties (OR = 2.4, 95% CI 1.5–4.8) in the area of D, high nursing hierarchy (OR = 2.7, 95% CI: 1.5–4.8), poor wages (OR = 2.9, 95% CI: 1.6–5,6), and too frequent night duties (OR = 2.3, 95% CI: 2.3–4.5) in the area of RPA. *Conclusions*. Prevalence of burnout among these nurses was high. The government therefore needs to look into factors that will enhance nurses' recruitment and retention for effective health care delivery system.

## 1. Introduction

The burnout syndrome is a serious consequence of chronic exposure to work-related stressors. The construct of burnout syndrome appeared for the first time around the early 1970s, aimed at explaining the process of physical and mental deterioration in professionals working in areas such as teaching, health care, social work, or emergency legal services [1]. Subsequently, burnout syndrome was defined as a sustained response to chronic work stress comprising three dimensions: the experience of being emotionally exhausted (emotional exhaustion), negative attitudes and feelings toward the recipients of the service (depersonalization), and feelings of low accomplishment and professional failure otherwise known as lack of personal accomplishment [2].

Among nurses, the incidence of occupational stress-related burnout is high and factors such as age of the nurse, years of service, hierarchy of nurse [3], lack of adequate staff, difficult or demanding patients [4], younger age, male gender, and inadequate clinical supervision [5] have been reported to be associated with burnout. Others are excess workload, emotional stress, unevaluated work and underpayment [6], poor leadership, death and dying, conflicts with staff, accepting responsibility, lack of social support, conflict with other nurses, conflict with physicians, presence of stressors related to private life, feeling that the job is threatened, and higher nursing grade [7].

Similarly, Kingdom [8] noted that excess workload and number of people living in the household were the best predictors of physical health problems among nurses in Japan, while likelihood to leave the current nursing position and lack of support in the workplace were the best predictors of mental problems. Stress is a factor that affects nurses on a daily basis and can result in nurses' absenteeism and aggression as well as reduced productivity and efficiency [8]. Burnout has a special significance in health care settings where staff experience both emotional and physical stress.

 A growing recognition of job stress leading to dissatisfaction among registered nurses in Nigerian hospitals has contributed to current problems with recruitment and retention of nurses in the country in general but more peculiar in some states. If nurse administrators identify factors influencing nurses' job satisfaction in government hospitals and implement strategies to address these factors, nurses' turnover rate will decrease and recruitment and retention rates will increase. Moreover, burnout among nurses has a negative effect on the quality of patient care [9, 10].

Several studies have demonstrated various components of burnout among nurses in developed countries of the world [7, 8, 11]. However, not much has been done in a poor resource, multiethnic, and culturally laden country like Nigeria. There is a dearth of information on this subject matter, hence the need for this study.

## 2. Study Area

The study took place at the State Hospital, Ring Road, Ibadan. Ibadan is the capital of Oyo state, Nigeria, and it is the third largest city in Nigeria. The city is located in the southwestern part of the country. It has a population of over 3.5 million people. Ibadan is divided into eleven local government areas [12]. The hospital is owned by the State Government and it attends to all medical, surgical, and psychiatric cases. There are two hundred and ninety-two (292) nurses working in the hospital. Skill mix is not practiced in this hospital. Patient flow is quite high cutting across all social strata.

## 3. Methodology

### 3.1. Data Collection

 There were two sources of data. The first was existing hospital records while the second was a survey. Ethical approval was obtained in February, 2008 from the ethical review committee of the Oyo State Ministry of Health. For the survey, individual letters were written to all nurses working at Ring Road State Hospital Ibadan in April, 2008, to inform and request for their participation in the study. A total sampling method was utilized. Consent was obtained from each participant and information obtained was made anonymous.

Information about the number of nurses and their status, attending doctors, coverage of all shifts by doctor on call, and patient-to-nurse ratio was obtained from the hospital documents.

## 4. Survey Instrument

The second document was a self-administered questionnaire divided into four parts. Part 1 included basic demographic data (age, sex, marital status, number of children, religion, and time to reach their hospital) and some questions about experiences during the past month (number of night shifts and conflict with other nurses or with doctors).

Part 2 consisted of the 12-item GHQ [13] and the Maslach Burnout Inventory (MBI) [14]. All survey instruments were individually handed over to the participants during a monthly staff screening exercise after each of them had received individual code only known to the research team members. This coding system enabled participants to be contacted after the study for those who required intervention.

The GHQ is a screening instrument for psychiatric morbidity. Although it does not yield a diagnosis, positive scores are indicative of psychological distress. Each item is rated either 0 or 1 on the basis of the frequency with which the subject has experienced the symptom in the recent past, yielding a maximum score of 12. A score of 1 or above is suggestive of psychological distress; however, to increase specificity, a cut-off point of 2 was used and score of 2 was used as positive screen.

Maslach Burnout Inventory (MBI) is a 22-item self-report inventory designed to measure the characteristics of burnout. It has a dual column response format. The frequency column has a 6-point Likert-type format and the intensity column is 7 points.

Direct scoring was used for the items of the Emotional Exhaustion (EE) and Depersonalization (D) Subscales by adding together the values of the ratings shaded while reverse scoring was used for the item of the Personal Accomplishment (P) Subscale by adding together the reversed values of the shaded ratings. Low score represents presence of burnout, while high score represents absence of burnout. All subsections were added together for overall score. The Maslach Burnout Inventory has cross-cultural reliability and validity, and, in Nigeria, a study aimed at establishing the psychometric properties of the MBI using a sample of health care workers obtained reliability coefficients as follows: Cronbach's alpha of 0.86 and split-alpha of 0.57 [15].

## 5. Pilot Test

These survey instruments were pilot tested among 30 nurses from Adeoyo Maternity Hospital, a sister hospital also being run by the same board as the study center. This was to assess the applicability of instruments and suitability of study design. The pilot study showed that the design of the study was appropriate to the study population.

## 6. Statistical Analysis

All statistics were performed with SPSS 13.0 software [16]. Initially, bivariate analyses compared certain demographic variables such as gender, age, and hierarchy with high burnout. Pearson's chi-square was used to compare categorical variables, while *t*-test was used to compare two continuous variables and two-way ANOVA for more than two continuous variables.

For multivariate risk analysis of high burnout variables that were significant during bivariate analysis were entered into the model of the logistic regression equation. Variables were entered in the binary form, that is, 0, 1, and were coded in a way to illustrate the effect of each level. To facilitate the interpretation of odds ratio, a reference category was always chosen for the independent variables with which other variables could be compared.

## 7. Results

A total of 270 of the 292 (92.3%) nurses participated.

### 7.1. Characteristics

Table 1 summarizes the characteristics of the respondents. They were 270 in all, 64.1% were women, all (100%) were practicing a religion, 70% were married, and only 10% were staff nurses. The mean age was 38.3 ± 19.9 years. One hundred and ten (40.7%) scored positive on GHQ.

### 7.2. Prevalence of Burnout

A high level of burnout was identified in 39.1% of the respondents in the area of emotional exhaustion (EE), 29.2% in the area of depersonalization, and 40.0% in the area of reduced personal accomplishment; this was significant *χ* ^2^ = 8.4 df (2) *P* = 0.01 (Table 2).

### 7.3. Prevalence of Burnout and Associated Factors

Older nurses (*P* < 0.01, female gender (*P* < 0.01), being unmarried (*P* < 0.01), junior nursing hierarchy (*P* < 0.01), nurse/doctor conflict (*P* < 0.01), and too frequent night duties (*P* < 0.01) were significantly associated with high burnout in all the three dimensions (Table 3).

Nonavailability of a doctor to work with (*P* < 0.02) was significantly associated with burnout in the area of emotional exhaustion and depersonalization and also in the area of reduced personal accomplishment (*P* < 0.01) (Table 3).

Poor wages were significantly associated with burnout in the area of emotional exhaustion (*P* < 0.02), in the area of depersonalization (*P* = 0.04), and in the area of reduced personal accomplishment (*P* = 0.01) (Table 3). 

### 7.4. Risk Factors for Burnout: Multivariate Analysis

Multivariate analysis showed that doctor/nurse conflict (OR = 3.1, 95% CI: 1.9–6.3), low doctor/nurse ratio (OR = 6.1, 95% CI: 2.5–13.2), inadequate nursing personnel (OR = 2.6, 95% CI: 1.5–5.1), and too frequent night duties (OR = 3.1, 95% CI: 1.7–5.6) were predictors of burnout in the area of emotional exhaustion and doctor/nurse conflict (OR = 3.4, 95% CI: 2.2–7.6), low doctor/nurse ratio (OR = 2.4, 95% CI: 1.4–4.1), and too frequent night duties (OR = 2.4, 95% CI: 1.5–4.8) in the area of depersonalization. High nursing hierarchy (OR = 2.7, 95% CI: 1.5–4.8), poor wages (OR = 2.9, 95% CI: 1.6–5,6), and too frequent night duties (OR = 2.3, 95% CI: 2.3–4.5) in the area of reduced personal accomplishment were associated with high burnout after model adjustment (Table 4).

## 8. Discussion

Burnout appears to be highly prevalent among nurses in this hospital. These subjects also have high prevalence of psychiatric morbidity. Our study has demonstrated a significant difference in the prevalence of burnout across the three dimensions. There were also demographic variations in the prevalence of burnout among these nurses across these burnout dimensions. These demographic differences were present for age, gender, and marital status. We found that high burnout was significantly reported by older nurses, a finding previously reported [17] but which contrasts an earlier one [5]. As previously reported [17–19], we also found that junior nursing hierarchy was significantly associated with high burnout. This appears paradoxical as the senior nurses are expected to have reported more burnout compare d with junior ones since the senior ones should be older in age. This result may not be unassociated with bullying in nursing which is fast becoming a common practice [20, 21]. More women reported high burnout in this study contrast to that of Edwards and colleagues [5] although the same has been reported even among other professional such as physicians [22].

Our study also suggests that marriage is a protective factor against high burnout. The present study was not able to corroborate an earlier one in Japan [8] which reported that number of people living in the household is the best predictor of burnout among nurses.

We found that nurse/doctor conflict and nonavailability of physicians to work with were associated with burnout. Stehle [23] similarly noted that many of the stressors identified among nurses concerned working relationships with doctors. The potential explanation for this association in the present study is that nonavailability of physicians to work with would require the nurses to pay additional attention to the patients in order to “cover up” for the doctor's absence thereby creating additional strain for them. This singular factor underscores the importance of team building in the prevention of burnout in nurses.

We did not find an association between burnout and number of children or other personal factors but found that organizational factors, too frequent night shifts, poor wages, and inadequate security at night, were associated with high burnout. One of the reasons why inadequate security at night could have been associated with high burnout is increased risk to physical injuries and assaults. For Maslach and colleagues [24], burnout is a response to overload and some of the factors retained after model adjustment during multivariate analysis were as a result of work overload. It has been suggested that reducing hours may be the first step to reduce resident burnout [25] and this is applicable to nurses.

In our multivariate analysis, as previously reported [26–28], all the predictors of burnout among the nurses were essentially organizational factors (doctor/nurse conflict, inadequate number of physicians to work with, inadequate nursing personnel, and too frequent night duties), suggesting the need for organizational restructuring of the health services of the Oyo state if prevalence of burnout among the nurses is to reduce. There is an increased likelihood for illness and injury among employees working in long-hour schedules and schedules involving unconventional shift work (e.g., night and evening shifts). These issues are of utmost ethical import taking into consideration fatigue-related errors such employees working in these kind of demanding schedules may be liable. Such errors could have serious and adverse repercussions for public safety [29]. Thus, the result from this study indicating the association between too frequent night duties and burnout is of utmost ethical consideration considering the medical errors that could accrue from such practice.

The prevalence of psychiatric morbidity reported in this study high is close to that of burnout in the areas of emotional exhaustion and reduced personal accomplishment illustrating that although burnout is not a recognized clinical psychiatric or psychological disorder, there are some similar features between burnout and diagnosable conditions such as depression, anxiety disorders, or mood disorders [30]. Similar high prevalence of psychiatric symptoms has been reported in other parts of the world among nurses [31, 32].

A major strength of this study is that this is the first study to our best knowledge of the largest nursing workforce in Nigeria. Although the study was limited to one of the hospitals being managed by an umbrella board, findings from this study may be generalized to other general hospitals in the state.

The present study has a number of potential limitations: our aim was to focus on the individuals (nurses) rather than on the patients themselves, thus patients' factors and interdepartmental differences were not included in the analysis. We also did not study various coping strategies employed by these nurses. We were not also able to correlate the GHQ score with level of burnout. These should be considered in future studies.

## Figures and Tables

**Table 1:** Demographics variables of all respondents.

Variable	No	Frequency (%)/mean
Number	270	100
Age, yr (mean ± SD)	—	38.3 ± 1 9.9
>mean	181	67.0
Women	173	64.1
Married or with a partner	189	70.0
Number of children ≥1	163	60.3
Practicing Christianity	141	52.2
Traveling time to work, min (mean ± SD)	—	35.9 ± 19.6
Status		
Chief Nursing Officer,	91	33.7
Assistant Chief Nursing Officer	72	26.7
Principal Nursing Officer	44	16.3
Senior Nursing Officer	31	11.5
Nursing Officer	22	8.2
Staff Nurse	10	3.7
Working hours per week, (mean ± SD)	—	48.2 ± 8.2
Night shift in days per month, mean ± SD	—	19.6 ±.11.4
>mean night shift in/month days	101	30.0
Mean shift allowance p/a in US dollars	—	240 ± 120
Mean total salary in US dollars	—	2400 ± 1800
Regularity of salary (Yes)	4	1.5
GHQ +	110	40.7

**Table 2:** Prevalence of burnout.

Emotional exhaustion (EE)			Depersonalization (D)			Reduced personal accomplishment		
*n* (%)	*n* (%)	*n* (%)	*n* (%)	*n* (%)	*n* (%)	*n* (%)	*n* (%)	*n* (%)
Low	Moderate	High	Low	Moderate	High	Low	Moderate	High
≤18	19–26	≥27	≤5	6–9	≥10	≥40	39–34	≤33
164 (60.9%)	61 (18.7%)	55 (20.4%)	191 (70.8%)	36 (13.3%)	43 (15.8%)	162 (60.0%)	45 (16.7%)	63 (23.3%)

	Burnout			Burnout			Burnout	
	(EE)			(D)			(RPA)	

No		Yes	No		Yes	No		Yes
*n* (%)		*n* (%)	*n* (%)		*n* (%)	*n* (%)		*n* (%)
164 (60.9)		106 (39.1)	191 (70.8)		79 (29.2%)	162 (60.0)		108 (40.0)

*χ* ^2^ = 8.2. *P* < 0.01.

**Table 3:** Sociodemographic characteristics and burnout.

Variable	*N*	EE	%	sig	D	%	sig	RPA	%	sig
		*n* = 106			*N* = 79			*N* = 108		
Age ≤38.3	51	7	9.8	<0.01	6	11.8	<0.01	7	13.7	<0.01
Age >38.3	219	99	45.2		71	32.4		101	46.1	
Male	97	27	27.8	<0.01	19	19.6	<0.01	26	26.8	<0.01
Female	173	79	64.2		60	34.7		82	47.4	
Married or with partners	189	37	19.6	<0.01	39	20.6	<0.01	33	17.5	<0.01
Unmarried	81	69	85.1		40	49.3		75	92.6	
No children	107	46	43.0	0.3	40	47.3	0.02	36	33.6	0.08
≥1 Children	163	60	36.8		39	23.9		72	44.2	
Traveling time to work (mins) ≤35.9	201	76	37.8	0.4	62	30.8	0.3	78	38.8	0.5
Traveling time to work (mins) >35.9	69	30	43.5		17	24.6		30	43.5	
Status: CNO/ACNO/PNO	205	70	34.1	<0.01	45	21.9	<0.01	70	34.1	<0.01
Status: SNO/NO/Staff Nurse	65	36	55.4		34	52.3		38	58.4	
Nurse/nurse role conflict (yes)	80	36	45.0	0.2	24	30.0	0.7	36	45.0	0.3
Nurse/nurse role conflict (no)	190	70	36.8		53	27.9		72	37.9	
Nurse/doctor role conflict (yes)	120	63	52.5	<0.01	45	37.5	<0.01	75	62.5	<0.01
Nurse/doctor role conflict (no)	150	43	28.7		34	22.7		33	22.0	
Availability of physicians to work with (yes)	34	7	20.6	<0.02	4	11.8	0.02	7	20.6	0.01
Availability of physicians to work with (no)	236	99	41.9		75	31.8		101	42.8	
Lack or inadequate nursing personnel (yes)	93	22	23.7	<0.01	17	18.3	<0.01	20	21.5	<0.01
Lack or inadequate nursing personnel (no)	177	84	47.5		62	35.0		88	49.7	
Poor Wages (yes)	249	103	41.4	<0.02	77	30.9	0.04	103	41.4	0.01
Poor Wages (no)	21	3	14.3		2	9.5		5	7.0	
Too frequent night duties (yes)	187	90	48.1	<0.01	70	37.4	<0.01	92	49.1	<0.01
Too frequent night duties (no)	83	16	19.2		9	10.8		16	19.2	
Inadequate security during night duty (yes)	260	104	40.0	0.2*	76	29.2	0.9*	106	40.8	0.2
Inadequate security during night duty (no)	10	2	20.0		3	30.0		2	20.0	

*Fisher's Exact.

EE: Emotional exhaustion; D: depersonalization; R: reduced Personal accomplishment.

**Table 4:** Significant predictors of burnout among nurses.

Variables	EE	Sig	D	Sig	RPA	Sig
Very senior nurses	NS		NS		2.7 (1.5–4.8)	0.03
Doctor/nurse role conflict	3.1 (1.9–6.3)	0.03	3.4 (2.2–7.6)	0.03	NS	
Inadequate number of physicians to work with	6.1 (2.5–13.2)	<0.01	2.4 (1.4–4.1)	0.03	NS	
Inadequate nursing personnel	2.6 (1.5–5.1)	0.03	NS		NS	
Poor (Wages)	NS		NS		2.9 (1.6–5.6)	0.03
Too frequent night duties	3.1 (1.7–5.6)	0.03	2.4 (1.5–4.8)	0.03	2.3 (1.4–4.5)	0.03

EE: emotional exhaustion; D: depersonalization; RPA: reduced personal accomplishmen.
